# The Cytokine Ciliary Neurotrophic Factor (CNTF) Activates Hypothalamic Urocortin-Expressing Neurons Both *In Vitro* and *In Vivo*

**DOI:** 10.1371/journal.pone.0061616

**Published:** 2013-04-23

**Authors:** Matthew J. Purser, Prasad S. Dalvi, Zi C. Wang, Denise D. Belsham

**Affiliations:** 1 Department of Physiology, Toronto General Hospital Research Institute, University Health Network, Toronto, Ontario, Canada; 2 Departments of Obstetrics, Gynaecology and Medicine, University of Toronto and Division of Cellular and Molecular Biology, Toronto General Hospital Research Institute, University Health Network, Toronto, Ontario, Canada; University of Rouen, France

## Abstract

Ciliary neurotrophic factor (CNTF) induces neurogenesis, reduces feeding, and induces weight loss. However, the central mechanisms by which CNTF acts are vague. We employed the mHypoE-20/2 line that endogenously expresses the CNTF receptor to examine the direct effects of CNTF on mRNA levels of urocortin-1, urocortin-2, agouti-related peptide, brain-derived neurotrophic factor, and neurotensin. We found that treatment of 10 ng/ml CNTF significantly increased only urocortin-1 mRNA by 1.84-fold at 48 h. We then performed intracerebroventricular injections of 0.5 mg/mL CNTF into mice, and examined its effects on urocortin-1 neurons post-exposure. Through double-label immunohistochemistry using specific antibodies against c-Fos and urocortin-1, we showed that central CNTF administration significantly activated urocortin-1 neurons in specific areas of the hypothalamus. Taken together, our studies point to a potential role for CNTF in regulating hypothalamic urocortin-1-expressing neurons to mediate its recognized effects on energy homeostasis, neuronal proliferaton/survival, and/or neurogenesis.

## Introduction

Ciliary neurotrophic factor (CNTF) is a member of the four-helix bundle cytokine family, and has been shown to enhance the survival and differentiation of neurons in the central and peripheral nervous system [1]. It is expressed mainly in glial cells, but also in neurons, and is thought to convey its cytoprotective effects through activated release after stress or injury. CNTF stimulates gene expression, cell survival or differentiation in a variety of neuronal cell types such as sensory, sympathetic, ciliary and motor neurons. For these reasons, CNTF has been used as a treatment for patients afflicted with the neurodegenerative disease amyotrophic lateral sclerosis (ALS), but unexpectedly, CNTF administration led to substantial weight loss by these subjects. This effect has been further analyzed in obese mice [2] and humans [3] and attributed to changes at the level of the hypothalamus [4]. In particular, the CNTF-mediated sustained reduction in body weight and appetite was attributed to induced plasticity and regeneration of hypothalamic neurons [2], although their phenotypes are not yet fully defined.

Structurally, the hypothalamus consists of an array of fully differentiated neurons regulating many vital functions that include energy homeostasis. The specific cell types and neuropeptides involved in these complex functions have been extensively investigated, however, little is known about the action of CNTF in specific neuropetidergic neurons. The hypothalamus is subdivided into several nuclei consisting of groups of neurons with specific functions. The arcuate nucleus (ARC), paraventricular nucleus (PVN), ventromedial nucleus (VMH), dorsomedial nucleus (DMH), lateral hypothalamus (LH), and periventricular hypothalamic nucleus (PeV) play an important role in the regulation of energy intake and expenditure by integrating central and peripheral orexigenic and anorexigenic signals. It is well established that there are two distinct neuronal populations in the ARC: neuropeptide Y (NPY)/agouti-related peptide (AgRP), and α-melanocyte stimulating hormone (MSH)/pro-opiomelanocortin (POMC) neurons. Anorexigenic urocortin-1 has been shown to be expressed in midbrain sympathetic neurons adjacent to the Edinger-Westphal nucleus of the brainstem [5], in the hypothalamus, pituitary, and substantia nigra. Although there is still some inconsistent data as to which exact nuclei in the hypothalamus express urocortin-1, it has been reported to be present in widespread areas of the hypothalamus, including the PVN, VMH, and DMH among others, in humans, primates, and rodents [6], [7], [8], [9], [10], [11], [12], [13].

The CNTF receptor (CNTFR) is a cytokine receptor that classically acts through JAK/STAT activation. However, in the hypothalamus the exact neurons activated by CNTF to induce anorexia and whether appetite-regulating neuropeptide gene expression is altered within CNTFR-expressing hypothalamic neurons remain to be determined. We propose that hypothalamic CNTFR activation by CNTF regulates feeding-related neurons, and modulates neuropeptide expression via JAK/STAT activation. Using *in vitro* and *in vivo* models, we studied the modulation of urocortin-1 gene expression following CNTF treatment, the signaling mechanisms involved and mapped the areas of hypothalamic neuronal activation by CNTF.

## Experimental Procedures

### Cell culture and reagents

mHypoE-20/2 neurons were grown in Dulbecco's modified Eagle medium (DMEM, Sigma, Canada), supplemented with 5% fetal bovine serum (Life Technologies (Invitrogen), Burlington, ON, Canada), and 1% penicillin/streptomycin (Life Technologies (Gibco), Burlington, ON, Canada), and maintained at 37°C in an atmosphere of 5% CO2 [14]. CNTF was obtained from R&D Systems, USA. 30 ng of CNTF in a total of 3 ml DMEM was used in the cell culture studies, which is equivalent to a 0.45 nM concentration. The mHypoE-20/2 neurons were chosen for analysis due to the expression of urocortin-1 and urocortin-2, AgRP, brain-derived neurotrophic factor (BDNF), and neurotensin.

### One-step reverse transcriptase-polymerase chain reaction (RT-PCR)

One-step RT-PCR was carried out to screen the cells for urocortin-1, urocortin-2 AgRP, BDNF, neurotensin and CNTFR genes using One-Step RT-PCR Kit (Qiagen, Inc., Toronto, ON, Canada). The primer pairs used are listed in Supplemental Table 1. Total RNA (200 ng) was used for all samples in a total reaction volume of 25 µL. The RT protocol used for all genes was 50°C for 30 min, 95°C for 15 min, followed by amplification using 95°C for 15 s, 60°C for 15 s and 72°C for 1 min (40 cycles) and final incubation for 7 min at 72°C. All PCR-amplified products were visualized on 2% agarose gels containing ethidium bromide under ultraviolet light. The identity of the PCR amplicons were verified by purification and sequencing (The Centre for Applied Genomics, Toronto, ON, Canada).

### Quantitative reverse transcription-polymerase chain reaction (qRT-PCR)

Cells were harvested at the indicated time points following treatment with vehicle or CNTF (10 ng/ml). Real-time qRT-PCR was performed with 2.0 µg of total RNA using SYBR green PCR master mix (Life Technologies (Applied Biosystems Inc.), Burlington, ON, Canada), and ran on the Applied Biosystems Prism 7000 real-time PCR machine [15]. The sequences of the SYBR primers for the neuropeptides (Ct values between 26–30) and histone 3a (Ct values between 20–22) are found in Table S1. Data were represented as Ct values, defined as the threshold cycle of PCR at which amplified product was first detected, and analyzed using ABI Prism 7000 SDS software package. Copy number of the amplified gene was standardized to histone 3a using the standard curve method. The final fold differences in mRNA expression were relative to the corresponding time-matched control.

### SDS-polyacrylamide gel electrophoresis and Western blot analysis

Protein from 80–90% confluent mHypoE-20/2 cells was prepared as described previously [16]. Protein concentration was determined using the BCA protein assay kit (Thermo Fisher Scientific, Waltham, MA, USA). Total protein (20 µg) was resolved on SDS-PAGE gels and blotted onto Immun-Blot polyvinyl difluoride membrane (Bio-Rad, Mississauga, ON, Canada). Primary antibodies used in Western blotting included: phospho-JAK2 (Tyr1007/1008; 1∶1000; Cell Signaling Technology), phospho-ACC (Ser79; 1∶1000; Cell Signaling Technology, through New England Biolabs, Pickering, ON, Canada), phospho-STAT3 (Tyr705; 1∶1000; Cell Signaling Technology), phospho-ERK1/2 (Thr202/Tyr204; 1∶1000; Cell Signaling Technology), phospho-Akt (Ser473; 1∶1000; Cell Signaling Technology), and G-protein β subunit (1∶5000; Santa Cruz Biotechnology Inc, Santa Cruz, CA). Secondary antibodies were purchased from Cell Signaling Technology, USA, and G-protein β subunit (1∶1000) antibody was purchased from Santa Cruz Biotechnology. Immunoreactive bands were visualized with horseradish peroxidase-labeled secondary anti-rabbit IgG (Cell Signaling Technology) at a 1∶5000 dilution and enhanced chemiluminescence (ECL Advance kit; GE Healthcare, USA). G-protein β subunit was used as a loading control.

### Animal experiments

Adult 2-month old male C57BL/6 mice (25–30 g) were housed individually in plastic rodent cages and maintained on a 12 h light/dark cycle with *ad libitum* access to conventional standard rodent chow and water. A 26-gauge stainless steel guide cannula (Plastics One, Roanoake, VA) projecting into the third cerebral ventricle was implanted into each mouse using flat-skull coordinates from bregma (anteroposterior −0.825 mm, mediolateral 0 mm, dorsoventral −4.8 mm), and correct cannula placement was verified as previously described [15], [17], [18]. Mice were allowed to recover for 7–14 days and were handled daily for at least one week prior to manipulations. All procedures were carried out in accordance with protocols and guidelines approved by the University of Toronto Animal Care Committee (protocol number 20007107).

### Intracerebroventricular (i.c.v.) microinjections for feeding study

For the animal treatments, CNTF was freshly dissolved in 0.9% saline and microinjected 1 h prior to the onset of the dark phase. Each mouse received either 1 µg CNTF or 0.9% saline in a total volume of 2 µl by slow infusion over 10–15 minutes through a 30-gauge needle (0.5 mg/ml). The mice were returned to their home cages with free access to a premeasured amount of chow and water, and the effect of i.c.v. CNTF on feeding was determined (4 mice per treatment group). In order to test the efficacy of the i.c.v. injections, we performed a short-term analysis of CNTF effects on food intake 1 h prior to the onset of the dark phase, as previously described for other anorexigenic neuropeptides [17], [18]. We found that i.c.v. CNTF significantly reduced food intake and body weight in *ad libitum*-fed wild type mice, and this effect lasted for at least 2 h (Fig. S1). The dose of CNTF used to induce anorexia was based on a previous report [2], and our results indicate that the CNTF dose used was effective and weight loss was comparable to that seen previously in mice [19].

### Assessment of neuronal activation by c-Fos immunohistochemistry (IHC)

To study the effect of acute administration of CNTF on the activation of hypothalamic neuropeptidergic neurons, *ad libitum* fed mice (4 mice per treatment group) were treated with either i.c.v. CNTF or 0.9% saline as described above for the feeding study. CNTF was administered one hour prior to the onset of dark cycle; c-Fos expression was assessed at two hours following the CNTF treatment and one hour into the dark cycle. Two h following i.c.v. injections, mice were anesthetized and perfused transcardially with ice-cold phosphate buffered saline followed by freshly prepared 4% paraformaldehyde solution. The brains were removed immediately, post-fixed in 4% paraformaldehyde, cryoprotected in sucrose, snap-frozen in an isopentane bath and stored at −80°C. Frozen brains were subsequently cut with a cryostat (Leica CM1510S, Leica Microsystems) in a rostral to caudal direction in the coronal plane into 20 µm sections, and serial sections were stored at −20°C in cryoprotectant.

The number of c-Fos- and urocortin-1 immunoreactive neurons in specific hypothalamic regions were quantitatively assessed. The specific primary antibodies and their concentrations used for detection of immunoreactivity are as follows: anti-c-Fos (1∶25,000; Calbiochem, Canada, Catalog # Anti-c-Fos (Ab-5) (4–17) Rabbit Antibody (PC38-100UL); rabbit anti-c-Fos against mouse, rat and human c-Fos; the antibody produces only nuclear staining), and anti-urocortin-1 (1∶1000; Phoenix Pharmaceuticals, Catalog No. H-031-31; rabbit anti-urocortin-1 against rat, human). The urocortin-1 antibody was validated for specificity by pre-adsorption with its immunizing peptide (Fig. S2). For the immunohistological analysis, every other section at 20 µm intervals through the hypothalamus was selected. The sections were allocated rostral to caudal to visualize the distribution of urocortin-1- or c-Fos-immunoreactive neurons on each hemisphere. Evenly spaced sections covering the region −0.70 mm to −1.94 mm from bregma were defined according to the Mouse Brain Atlas of Paxinos and Franklin [20]. The detection of c-Fos and urocortin-1 immunoreactivity was performed by conventional avidin-biotin-immunoperoxidase method using diaminobenzidine (DAB) as chromogen (Vectastain ABC Elite Kit; Vector Laboratories, Canada) [21]. We processed brain sections for immunohistochemical staining by Tyramide Signal Amplification method (TSA; PerkinElmer). Immunohistochemistry for c-Fos was performed with DAB to yield a brown nuclear reaction product, whereas immunohistochemistry for urocortin-1 was performed using DAB with Metal Enhancer (cobalt chloride), yielding a more intense blue/black cytoplasmic reaction product (Sigma, Catalog No. D0426).

Immunostained sections were examined under a Zeiss Axioplan 2 microscope outfitted with an AxioCam HRc camera and AXIOVISION 4.2 imaging software, as previously described [17], [18]. For the quantification of cells, every second section throughout the ARC, PVN, LH, DMH, PeV and VMH was taken to visualize the distribution of urocortin neurons throughout these nuclei (total 3–4 sections/mouse). For each of the ARC, PVN, LH, DMH, PeV and VMH from both hemispheres, an image was captured in a single plane of focus at ×40 magnification and a 0.2 mm^2^ box was placed in the center of the selected hypothalamic regions. Cells with brown nuclear staining were considered c-Fos-immunoreactive, whereas cells with dark blue/black cytoplasmic staining were considered urocortin-1 positive. The immunoreactive neuronal cells from both hemispheres of 4 to 6 sections per each animal were counted in a blind manner, and in each group the mean value of the cell counts per section of an individual animal was used for statistical analysis. The results were expressed as the ratio of cells co-expressing c-Fos with urocortin-1 to the total number of respective neuropeptide-immunoreactive cells per 0.2 mm^2^ area of the ARC, PVN, LH, DMH, VMH, or PeV.

### Statistical analysis

Data were analyzed using GraphPad Prism software (GraphPad Software, Inc., San Diego, CA, USA). Statistical analysis was performed using one-way or two-way ANOVA, and statistical significance was determined by post hoc analysis using Bonferroni test or Student's *t*-test with *P*<0.05.

## Results

### Expression of CNTFR and appetite-regulating neuropeptides in mHypoE-20/2

**Figure 1:**
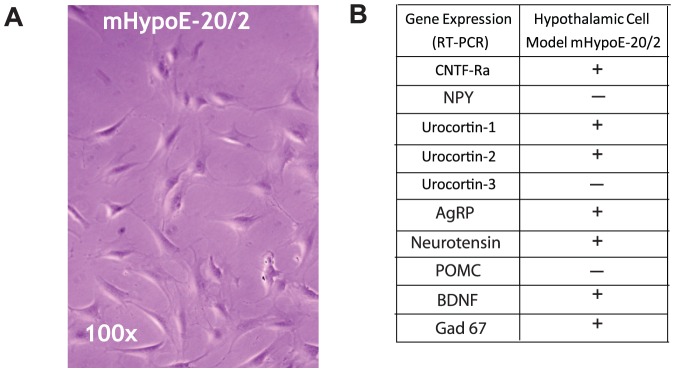
Expression profile of CNTF receptor and appetite-regulating neuropeptides in the mHypoE-20/2 neuronal cell line. (A) mHypoE-20/2 neurons were imaged using a phase contrast microscope at 100× magnification. (B) RNA harvested from mHypoE-20/2 neurons and hypothalamus control was used as a template for semi-quantitative RT-PCR. Listed in the table is the presence (+) or absence (−) of specific genes, including NPY, neuropeptide Y; AgRP, agouti-related peptide; POMC, proopiomelanocortin; urocortin-1; urocortin-2; neurotensin; BDNF, brain-derived neurotrophic factor; Gad 67, glutamate decarboxylase 67; CNTFRa, ciliary neurotrophic factor receptor alpha.

We have previously reported the generation and characterization of embryonic and adult mouse hypothalamic cell lines [14], [15]. We initially investigated whether our hypothalamic cell models developed and characterized in our laboratory contained CNTFR. These cell lines have been used for analysis of diverse physiological pathways, including leptin, insulin, and CNTF signal transduction [15], [22], [23], [24], as well as neuropeptide gene regulation and secretion [25], [26], [27]. Morphologically, the mHypoE-20/2 cell line is neuronal and expresses classic neuronal markers (Fig. 1A–B). Using RT-PCR we confirmed the presence of CNTFR mRNA in mHypoE-20/2 neuronal cell models (Fig. 1B). We also analyzed the expression of hypothalamic neuropeptides involved in appetite regulation in these cell lines to determine the best model for this study. The mHypoE-20/2 neurons were found to express high levels of CNTFR and other neuropeptides that were linked to feeding regulation (Fig. 1B), and was therefore used as the *in vitro* model.

### Activation of STAT3 by CNTF in the hypothalamic neuronal cells

**Figure 2:**
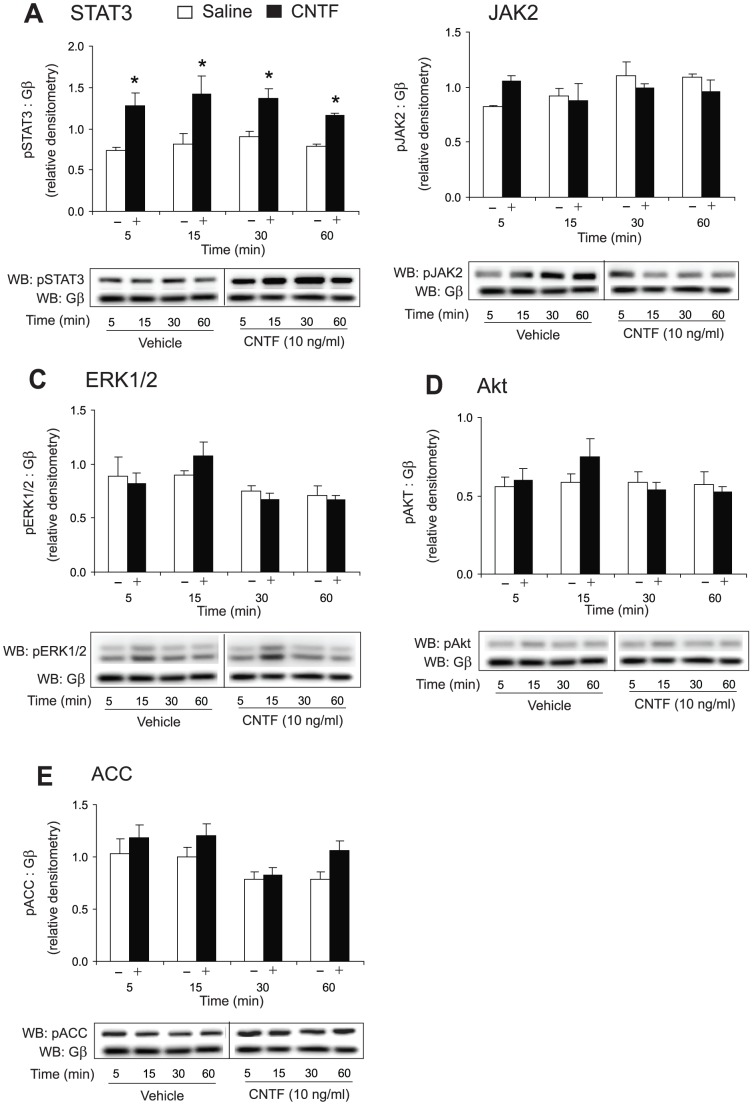
Effect of CNTF on phosphorylation of signaling proteins in the mHypoE-20/2 neuronal cells. (A–E) mHypoE-20/2 neurons were serum starved for 12–16 h before treatment with 10 ng/ml (0.45 nM) CNTF (+) or vehicle alone (−) over a 60 min time course. Western blot analysis of cell lysates was performed using phospho-specific antibodies directed against (A) STAT3, (B) JAK2, (C) ERK1/2, (D) Akt, and (E) ACC. All results shown are normalized to G-protein β subunit relative to corresponding control protein levels at each time point and are expressed as mean ± SEM (n = 4 independent experiments, *P<0.05). Representative Western blots are shown.

Given the key signaling cascade that CNTF activates upon binding to the CNTFR is the JAK/STAT pathway, we next determined CNTF-dependent JAK/STAT activation in the mHypoE-2/20 hypothalamic cell line. We investigated potential regulation of non-classical signaling cascades by assessing Akt, ACC, and ERK1/2 phosphorylation upon CNTF treatment, as well. The neuronal cells were treated with 10 ng/ml CNTF, and activation of STAT3, JAK2, ERK1/2, Akt, and ACC was analyzed over 60 min. By Western blot analysis, we found that CNTF significantly induced STAT3 activation in the mHypoE-20/2 cell line from 5–60 min (Fig. 2A). Further, CNTF was found to partially activate JAK2 at 5 min, but this did not reach significance, likely due to high basal levels of JAK2 activation in the cell line (Fig. 2B). ERK1/2, Akt, and ACC were not significantly changed over the 60 min timecourse (Fig. 2C–E).

### Regulation of urocortin-1 mRNA transcript expression by CNTF

**Figure 3:**
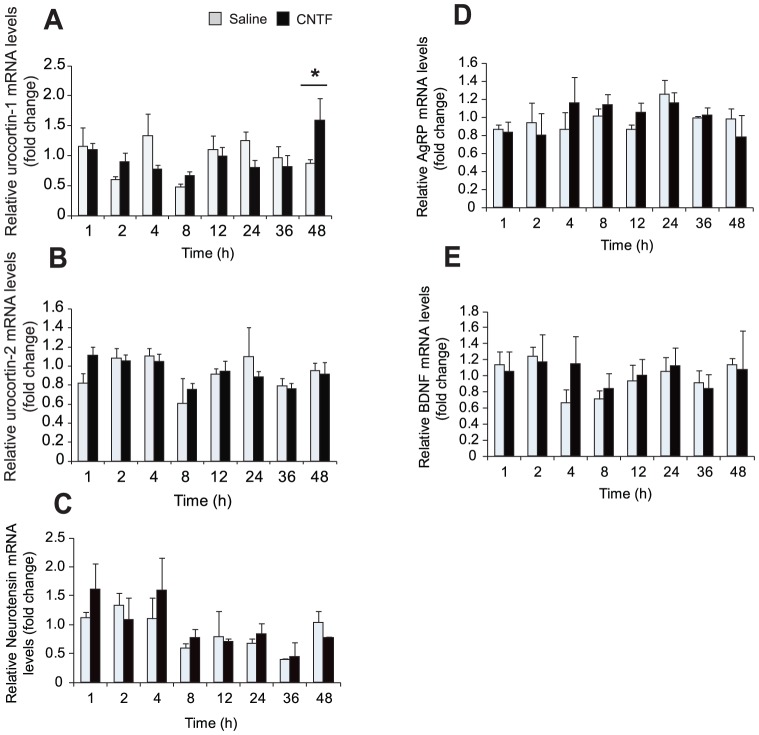
Effect of CNTF on regulation of neuropeptides linked to feeding in mHypoE-20/2 neuronal cells. Regulation of (A) urocortin-1, (B) urocortin-2, (C) neurotensin, (D) AgRP, and (E) BDNF mRNA expression by CNTF in the mHypoE-20/2 neuronal cell line. mHypoE-20/2 neuronal cells were exposed to 10 ng/ml (0.45 nM) CNTF, and real-time RT-PCR was performed over a 48 h timecourse. At the indicated time points, total RNA was extracted and used as a template for real-time RT-PCR with primers specifically designed to amplify each mRNA. mRNA levels were quantified using the standard curve method and normalized to the internal control (γ-actin). All results shown are relative to corresponding control mRNA levels at each time point. Data are represented as mean ± SEM (n = 5; *P<0.05; **P<0.01; ***P<0.001).

Next, we determined whether CNTF regulates mRNA transcript levels of a set of neuropeptides linked to energy homeostasis, including urocortin-1, urocortin-2, AgRP, BDNF, and neurotensin in the mHypoE-20/2 cell model. The hypothalamic neuronal cells were exposed to 10 ng/ml CNTF over a 48 h time course. Using real-time qRT-PCR, we found that in the mHypoE-20/2 neurons urocortin-1 mRNA expression was increased by 1.84-fold at 48 h (Fig. 3A). No change occurred in the transcript levels of the remaining neuropeptides upon CNTF treatment over vehicle control during the 48 h timecourse (Fig 3B–E). These results indicate that urocortin-1 is regulated by CNTFR activation in this hypothalamic neuronal model, and may be a potential downstream mediator of the effects of CNTF in the hypothalamus. However, in order to substantiate this claim, further analysis of the *in vivo* activation of these specific neuropeptide-expressing neurons would have to be delineated.

### Effect of i.c.v. CNTF on activation of hypothalamic nuclei

**Figure 4:**
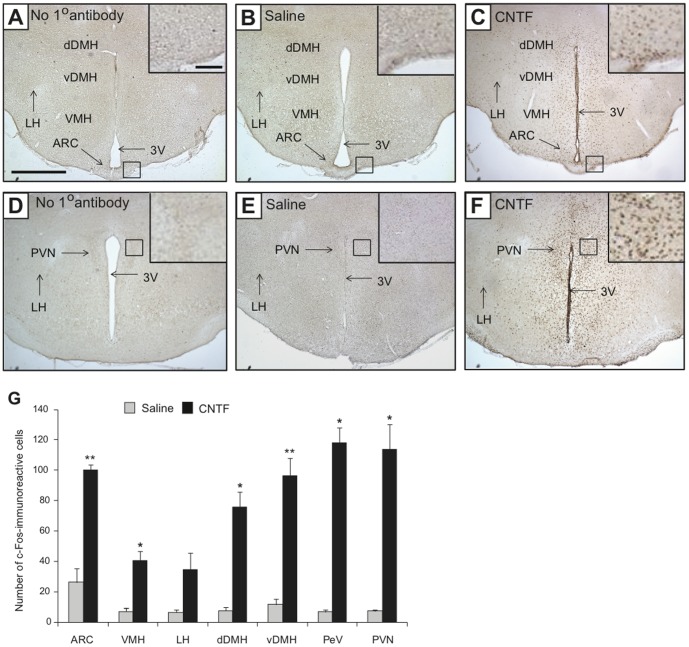
Acute CNTF treatment activates hypothalamic neurons. Immunohistochemistry was performed to assess neuronal activation by c-Fos-immunoreactivity (ir) in wild-type mice treated with intracerebroventricular (i.c.v.) saline or CNTF (0.5 µg/ml). A–F: Representative photomicrographs showing expression of c-Fos-ir in the hypothalamic ARC, VMH, LH, dDMH, vDMH, PeV, and PVN regions in coronal sections of mouse hypothalami (as indicated on the images). Scale bar: 1 mm. Inset in each image represents a higher magnification of the boxed area. Scale bar: 100 µm. A and D represent negative control images for the anti-c-Fos antibody. DAB staining: nuclear brown (c-Fos). 3V, third ventricle. G: Bar graph showing the number of c-Fos-ir neurons in the hypothalamic regions at 2 h post-treatment. Data in the bar graph are expressed as mean ± SEM (n = 4 animals/group; *P<0.05; **P<0.01).

As assessed by immunohistochemistry for c-Fos, a protein encoded by the immediate-early gene c-*fos*, we noted a remarkable level of neuronal activation in all areas of the hypothalamus after the central injection of CNTF compared with the saline controls (Fig. 4B, C, E, and F). This analysis was done 2 h post-injection, a time when c-Fos protein levels would be maximal. Significant increases in the number of c-Fos-positive neurons were detected in the hypothalamic ARC (increase by 3.65-fold), VMH (increase by 6.19-fold), LH (increase by 6.28-fold), dorsal DMH (dDMH) (increase by 10.13-fold), ventral DMH (vDMH) (increase by 9.29-fold), PVN (increase by 20.14-fold), and PeV (increase by 15.84-fold) (Fig. 4G). Activation of these hypothalamic regions was expected, as these regions widely express CNTFRs [1], [28], [29].

### Effect of i.c.v. CNTF on activation of urocortin-1 neurons

**Figure 5:**
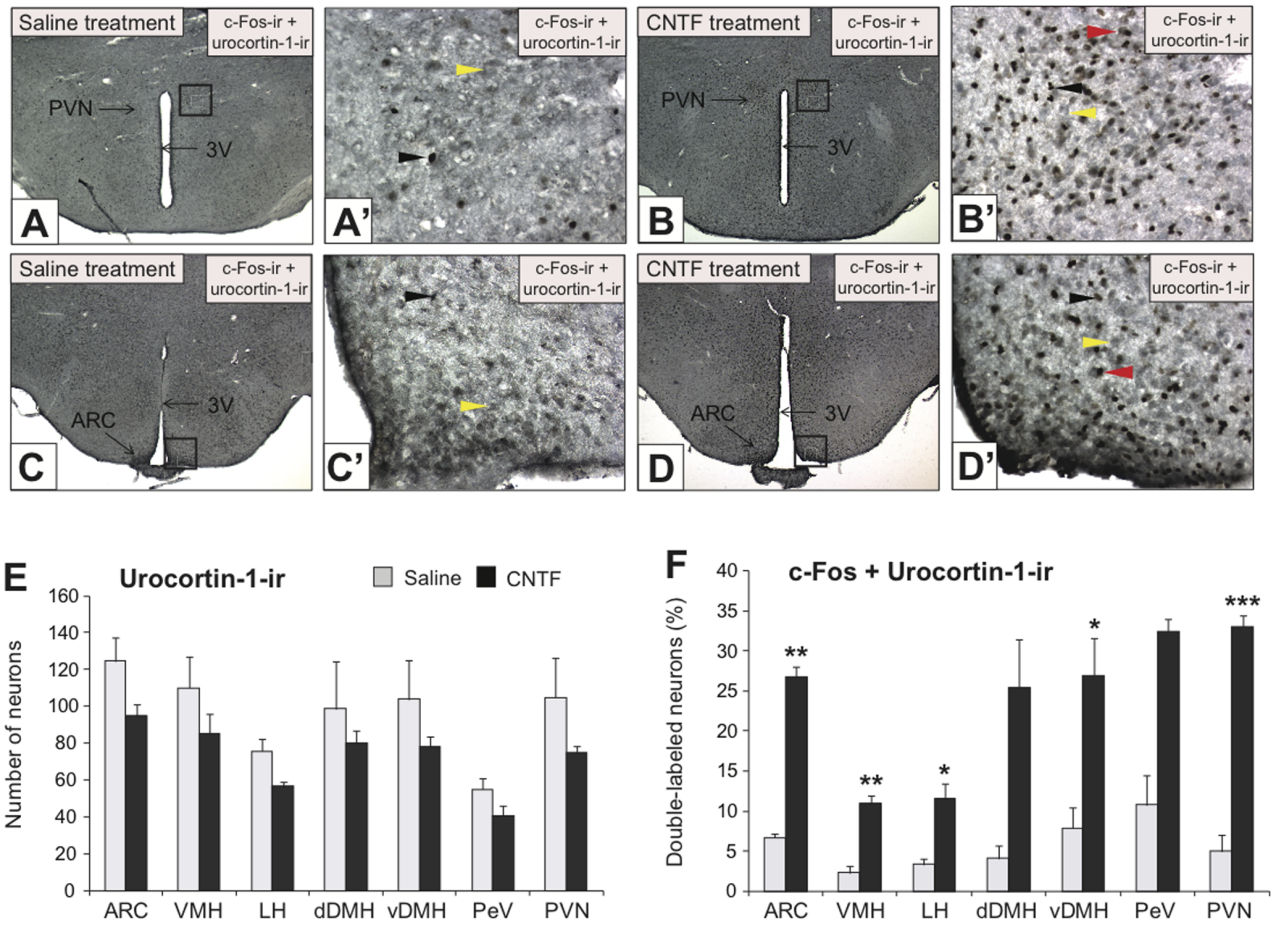
Acute CNTF treatment increases the number of hypothalamic neurons co-expressing c-Fos-immunoreactivity (ir) with urocortin-1-ir. Representative photomicrographs are shown of neurons co-expressing c-Fos-ir with (A–D) urocortin-1-ir in coronal sections of the hypothalami from wild-type mice at 2 h following i.c.v. administration of saline or CNTF (0.5 µg/ml). A–D: low magnification (×50) images of the hypothalamic regions (scale bar: 1 mm); A′–D′: High magnification (×400) images representative of the selected regions in the images A–D, respectively (scale bar: 100 µm). A–D: Co-expression of c-Fos-ir with urocortin-1-ir in the PVN (A, B) and ARC (C, D). Black arrowheads represent neurons expressing only nuclear c-Fos-ir, yellow arrowheads represent neurons expressing only cytoplasmic perinuclear neuropeptide-ir, and red arrowheads represent double-labeled neurons with co-expression of c-Fos-ir and neuropeptide-ir. (E–F) Graphical representation showing the number of neurons expressing c-Fos and neuropeptide-immunoreactivity (ir) in the ARC, VMH, LH, dDMH, vDMH, PeV and PVN of the hypothalami from wild-type mice at 2 h following i.c.v. administration of saline or CNTF (0.5 µg/ml). Double-labeled immunohistochemistry for (F) c-Fos-ir and urocortin-1-ir indicates that intracerebroventricular CNTF activates hypothalamic neuropeptidergic neurons. No significant change in the number of neurons expressing only (E) urocortin-1-ir was observed in the hypothalamic regions of saline- or CNTF-treated animals. Data are represented as mean ± SEM (n = 4 mice/group; *P<0.05; **P<0.01; ***P<0.001 vs. saline treatment).

Our next goal was to determine the neuropeptidergic neurons activated in these regions by performing double-label immunohistochemistry for c-Fos and neuropeptide co-expression (Fig. 5). We focused our attention on urocortin-1, as this neuropeptide was highly expressed in our hypothalamic cell model and directly regulated by CNTF (as demonstrated by our *in vitro* experiments). Basal levels of urocortin-1 were not changed between saline and CNTF treatment 2 h post-i.c.v. injection (Fig. 5A, C, and E), indicating that basal levels of the neuropeptides is not changed by CNTF at the 2 h timepoint. However, when double-label immunohistochemistry was compared, we found that CNTF significantly activated urocortin-1-expressing neurons in the hypothalamus (Fig. 5B, D, and F). This does not indicate that there is a specific regulation of the neuropeptides, but instead that CNTF potentially affects these neurons *in vivo*. Urocortin-1 neurons were activated by CNTF in the hypothalamic ARC (increase by 3.99-fold), VMH (increase by 4.77-fold), LH (increase by 3.43-fold), vDMH (increase by 3.42-fold), and PVN (increase by 6.66-fold), but did not reach significance in the dDMH or PeV (Fig. 5F). These findings suggest that some of the specific roles of CNTF in the hypothalamus, such as energy balance and cellular proliferation, may be modulated by urocortin-1-expressing neurons in the hypothalamus.

## Discussion

The hypothalamus is a complex area of the brain that controls much of basic physiology, including energy homeostasis, stress, and reproduction, among other homeostatic systems. The hypothalamus is divided into many distinct nuclei that contain neurons mainly defined by their synthesis of specific neuropeptides, neurotransmitters, or receptors. These neurons are not generally found in single groups or clusters, but are dispersed throughout individual nuclei of the hypothalamus. Due to this heterogeneity, it is difficult to study the direct effects of any specific agent on a single neuronal cell type. To circumvent this issue, we have generated clonal, immortalized cell lines representing many unique phenotypic profiles from the hypothalamus [14], [15]. These cell models can be used to determine the molecular events involved in the direct regulation of neurons by specific signals from the periphery or even adjacent cells, including CNTF produced by glial cells upon insult, such as stress or immune responses. The mHypoE-20/2 was one cell line that was found to express the CNTFR, and also to synthesize a number of neuropeptides linked to feeding. Using the embryonic mHypoE-20/2 hypothalamic neuronal cell model, we found that CNTF increased urocortin-1 mRNA expression through a STAT-dependent mechanism. The other neuropeptides studied did not change over a 48 h timecourse, indicating that a number of the more “classic” neuropeptides, such as AgRP and neurotensin, may not be directly regulated by CNTFR activation, and may be altered through afferent neuronal activation. Interestingly, a recent study has also found that leptin activated urocortin-1 neurons in the midbrain through activation of the JAK-STAT pathway [30], suggesting that STAT3 may be directly involved in urocortin-1 gene regulation. Overall, our *in vivo* findings suggest that complex interactions may occur between satiety- and hunger-related neuropeptides in one or more hypothalamic nuclei to mediate the overall anorexic action of CNTF. Our *in vitro* findings suggest that regulation of hypothalamic urocortin-1 may lie downstream of CNTF neuronal activation, thus we pursued these specific neurons *in vivo* with the use of double-label immunohistochemistry.

CNTF has been shown to regulate food intake [4], [19], [31], [32], [33], [34] and neurogenesis [2], [35], [36] via receptor-dependent mechanisms. CNTF is produced by glial cells, as well as some neurons, to exert a neuroprotective role after various causes of nerve injury or stresses. CNTF has recently been found to be endogenously expressed in neurons and glia, and this endogenous expression has been postulated to be involved in the regulation of energy homeostasis and also the neuropeptides involved in this process [37]. Previous studies have indicated that both NPY and POMC synthesis could be regulated by CNTF [31], [38], [39], although it was unclear whether this regulation is direct or through afferent control mechanisms. A recent study found that CNTF appears to regulate POMC, and not NPY, gene expression by direct action on signaling within the nucleus itself [40]. Interestingly, the neurogenesis study by Kokoeva et al., identified the new neurons induced by CNTF expressed NPY and POMC, but the involvement of these two neuropeptides in the weight-regulating effects of CNTF were not immediately clear [2]. However, other than these two classical neuropeptide systems, we found that CNTF, at a dose that induced anorexia, activated hypothalamic ARC, LH, PVN, DMH and PeV (regions that widely express CNTFRs along with several neuropeptides involved in energy metabolism). Furthermore, using double-label immunohistochemistry, we detected that central CNTF significantly activated urocortin-1-expressing neurons in the ARC, LH, VMH, DMH, and PVN. The overall numbers of neurons in the hypothalamus that express urocortin-1 are relatively low, which reflects the previous reports of limited expression of urocortin-1 outside of the supraoptic nucleus or midbrain sympathetic neurons adjacent to the Edinger-Westphal nucleus of the brainstem [6], [7], [8], [9], [10], [11], [12], [13]. Nonetheless, this is in accordance with the specific areas defined in the hypothalamus that undergo plasticity through neurogenesis, which is hypothesized to be a control mechanism to overcome the detrimental effects of a high fat diet and obesity [2], [35], [41].

The specific cell types and neuropeptides involved in the complex interactions related to obesity have been extensively investigated in hypothalamic cultures, slices, and transgenic mice. However, little is known about the potential for hypothalamic neurogenesis in specific cell types. Until recently it was thought that neurogenesis in the adult brain was limited to the hippocampal dentate gyrus region, subventricular zone lining the lateral ventricles, and olfactory bulb [42], but recent reports indicate that the hypothalamus also possesses neuroproliferative potency [2], [35], [43], [44]. Kokoeva et al. demonstrated that the hypothalamus is capable of low levels of sustained neurogenesis that can be augmented by CNTF administration [2], [35]. The downstream effectors/factors responsible for CNTF-induced *de novo* generation of neurons are not yet identified. Using CNTF, we have shown that neurogenesis occurs in adult hypothalamic primary cell cultures as well, and the cell division then facilitates the introduction of the immortalization factor T-antigen through retroviral infection [15]. Our analysis indicates that most of the neurons isolated in this manner express a wide variety of neuropeptides linked to feeding, but always in unique phenotypic combinations [15]. Similarly, analysis of the specific neuronal cell types that can undergo neurogenesis *in vivo* (although currently somewhat limited due to the labour-intensive double-label studies required), indicates that many different neuropeptidergic neurons can be induced to proliferate in the hypothalamus under select conditions, but the exact phenotypes of these neurons are not yet defined [2], [35], [41].

The involvement of corticotropin-releasing factor (CRF) and the urocortin family of neuropeptides in feeding regulation and energy homeostasis has been established mainly through exposure of specific brain regions to these peptides [45], [46], [47], [48], [49]. However, the exact mechanisms by which CRF and urocortins exert their dramatic effects are not yet fully understood. CRF has been shown to decrease feeding through specific CRF receptors [50], [51], [52]. The picture on the urocortin family of peptides, on the other hand, is still coming into focus and much less is known about these peptides in terms of their overall function, and the mechanism underlying their role in energy homeostasis [53]. Urocortin-1, homologous to CRF and urotensin, was the first family member to be discovered [12]. It was found to bind with higher affinity to the CRF-R2, and potently released ACTH as well as the other classically known stress-related functions of CRF, but importantly was also linked to the repression of feeding [46], [47], [54]. Similarly, urocortin-2 and -3 were isolated and found to regulate feeding behaviour [45], [55], [56], [57], [58]. The regulation of feeding by all three urocortins has been linked to a CRF type 2 receptor mechanism. Exactly which neurons are responsible for the effect on feeding are not yet understood, however, studies focusing on the Edinger-Westphal nucleus of the brainstem support a role for urocortin-1 in energy homeostasis [59], [60], [61]. We found that only urocortin-1 was regulated by CNTF in the mHypoE-20/2 neuronal model, as urocortin-2 was not changed over a 48 h timecourse. The change in urocortin-1 gene expression occurred over the longer-term, at 48 h, indicating that CNTF may have intermediate signaling components that result in the regulation of urocortin-1. This regulation may be related to its role in inhibiting the orexigenic action of ghrelin and neuropeptide-Y [62]. Nevertheless, the direct action of CNTF at the level of the hypothalamus will likely be more complex than a single gene effect, and may involve a cascade of events resulting in the cessation of feeding and a decrease in body weight. For example, using another immortalized hypothalamic neuronal cell model derived from adult mouse, we have previously shown that acute CNTF induces proglucagon gene expression [15]. Moreover, we have detected that chronic CNTF upregulates proglucagon expression within hypothalamic neurons to induce neurogenesis that may be involved in regulation of energy homeostasis [15]. This scenario fits quite well with the overall action of CNTF in the long-term change in body weight, as seen in patients taking CNTF agonists over a treatment period [3].

One should use some caution with over-interpretation of the results in our study however, since we have currently shown that uorcortin-1 neurons can be activated by CNTF. It is difficult to directly correlate the temporal activation of these neuropeptides *in vivo* without a more detailed timecourse, which is obviously not technically feasible using ICC. There is a possibility that neuropeptide synthesis and secretion may be affected at early time points *in vivo* or *in vitro* by CNTF that needs to be investigated further by using sensitive detection methods. Unfortunately, currently available EIA, ELISA or RIA kits are not sensitive enough to detect urocortin-1 content secreted in very low (probably picomolar or lower) concentrations by the hypothalamic neuronal cell model. Even Western blot analysis is difficult due to the small size of the urocortin-1 peptide. Further analysis of the neuronal circuits involved in the overall anorexigenic effects of CNTF is essential to delineate the role of these neurons in CNTF action, perhaps using more sophisticated mouse models. Nonetheless, the role of urocortin-1 in this process is quite intriguing, since so little is known of the downstream role of urocortin-1 in energy homeostasis.

In summary, our data demonstrate that CNTF activates multiple hypothalamic sites where complex interactions may occur between appetite-regulating neuropeptidergic neurons to alter energy homeostasis and/or neuronal proliferation/survival. The findings from our *in vitro* model on the action of CNTF on urocortin-1 gene regulation complement our *in vivo* findings that these neurons can be activated and regulated by central CNTF. Ultimately, this study provides a previously unrecognized link between CNTF action and direct regulation of in these specific hypothalamic neuropeptides. The exact role of urocortin-1 in mediating the downstream effects of CNTF, as well as the afferent neurons involved, have yet to be delineated. However, it appears that CNTF activates specific neuronal networks in the hypothalamus to achieve its diverse regulatory roles on feeding and/or cell proliferation.

## Supporting Information

Figure S1 **Intracerebroventricular (i.c.v.) injection of CNTF inhibits food and water intake, and induces weight loss in wild-type mice.** To determine the efficacy of i.c.v. CNTF to induce anorexia, ad libitum-fed mice received injection of 1 µg of CNTF dissolved in 2 l of 0.9% normal saline 1 h before the onset of the dark cycle (0.5 mg/ml). Mice were returned to their home cages with pre-weighed amount of chow and water. Changes in (A) food, (B) water intake, and (C) animal weight were measured at 1 and 2 h postinjection. 0.9% normal saline solution was used as control treatment. All results are expressed as mean ± SEM (n = 4 mice/group; *P<0.05 vs. saline).
(PDF)

Figure S2 **Analysis of the specificity of the uorcortin-1 antibody after pre-adsorption with the immunizing peptide.** Immunocytochemistry was performed on the mHypoE-20/2 neurons and captured using a confocal laser microscope to assess the antibody specificity. Cells were incubated with urocortin antibody (+), preblocked antibody, or vehicle (−), and signals were amplified using a fluorescent conjugated secondary antibody. Nuclear staining was utilized to provide reference to staining localization.
(PDF)

Table S1 **Primer sequences used in the manuscript.**
(PDF)
