# Supplementary material for: The Cytokine Ciliary Neurotrophic Factor (CNTF) Activates Hypothalamic Urocortin-Expressing Neurons Both In Vitro and In Vivo
Source: PLoS One. 2013 Apr 23;8(4):e61616. doi: 10.1371/journal.pone.0061616 (PMC3633986; doi:10.1371/journal.pone.0061616)
Supplement: Figure S1 — Intracerebroventricular (i.c.v.) injection of CNTF inhibits food and water intake, and induces weight loss in wild-type mice. To determine the efficacy of i.c.v. CNTF to induce anorexia, ad libitum-fed mice received injection of 1 µg of CNTF dissolved in 2 l of 0.9% normal saline 1 h before the onset of the dark cycle (0.5 mg/ml). Mice were returned to their home cages with pre-weighed amount of chow and water. Changes in (A) food, (B) water intake, and (C) animal weight were measured at 1 and 2 h postinjection. 0.9% normal saline solution was used as control treatment. All results are expressed as mean ± SEM (n = 4 mice/group; *P<0.05 vs. saline). (PDF) [file pone.0061616.s001.pdf]

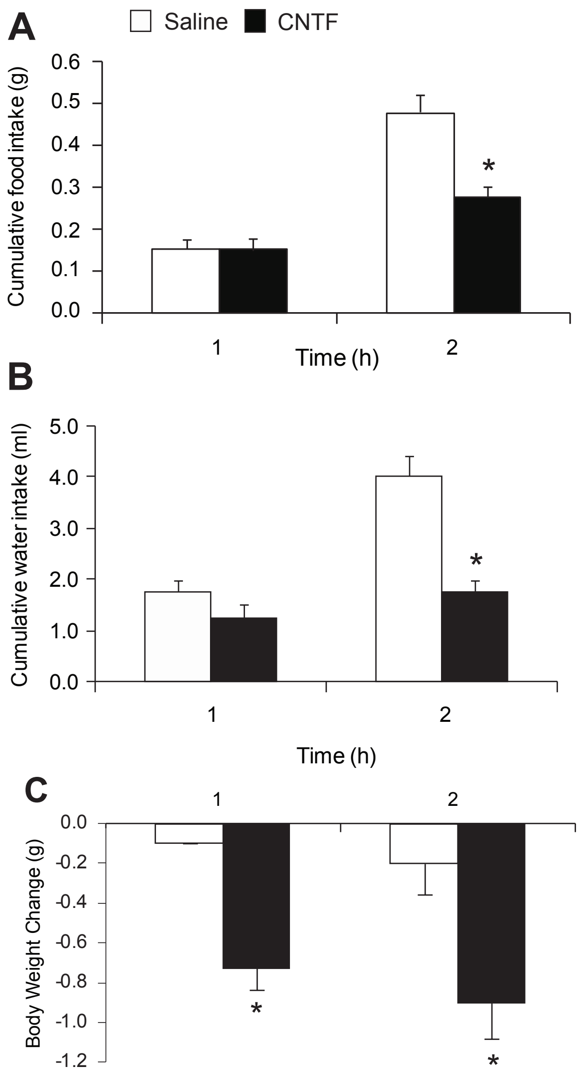

Supplem entary Fig ure 1- Purser et al.

Intracerebrove ntri cular (i.c.v.) injecti on of CNTF in hibits fo od and water intake, and induces wei ght loss in wi ld-type mic e. To det ermine the efficacy of i.c.v. CNTF to induce anorexia, ad libitum-fed mice received injection of 1 µg of CNTF dissolved in 2 µl of 0.9% normal saline 1 h before the onset of the dark cycle (0.5 mg/ml). Mice were returned to their home cages with pre-weighed amount of chow and water. Changes in (A) food, (B) water intake, and (C) animal weight were measured at 1 and 2 h post-injection. 0.9% normal saline solution was used as control treatment. All results are expressed as mean ± SEM (n = 4 mice/group; *P < 0.05 vs. saline).
