# Supplementary material for: The Cytokine Ciliary Neurotrophic Factor (CNTF) Activates Hypothalamic Urocortin-Expressing Neurons Both In Vitro and In Vivo
Source: PLoS One. 2013 Apr 23;8(4):e61616. doi: 10.1371/journal.pone.0061616 (PMC3633986; doi:10.1371/journal.pone.0061616)
Supplement: Figure S2 — Analysis of the specificity of the uorcortin-1 antibody after pre-adsorption with the immunizing peptide. Immunocytochemistry was performed on the mHypoE-20/2 neurons and captured using a confocal laser microscope to assess the antibody specificity. Cells were incubated with urocortin antibody (+), preblocked antibody, or vehicle (−), and signals were amplified using a fluorescent conjugated secondary antibody. Nuclear staining was utilized to provide reference to staining localization. (PDF) [file pone.0061616.s002.pdf]

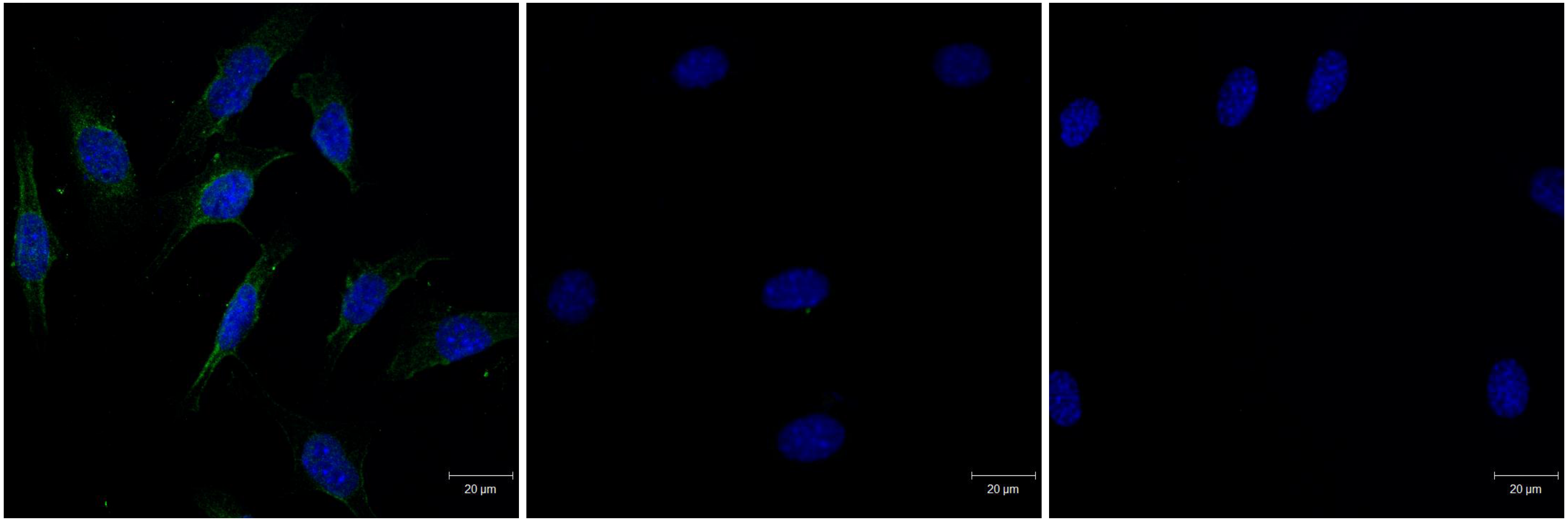

Urocortin Antibody

Urocortin Antibody-Peptide Preblocked

Primary Antibody Negative

Supplementary Figure 2 - Purser et al.
Analysis of the Specificity of the Uorcortin-1 Antibody after Pre-adsorption with Immunizing Peptide.
Immunocytochemistry was performed on the mHypoE-20/2 neurons and captured using a confocal laser microscope to assess the antibody specificity. Cells were incubated with Urocortin antibody (+), preblocked antibody, or vehicle (-), and signals were amplified using a fluorescent conjugated secondary. antibody Nuclear staining was utilized to provide reference to staining localization.

Supplementary Methods - Supplementary Figure 2 - Purser et al.
Preblock was performed with approximately 50:1 Molar ratio of peptide to antibody (as recommended by Pheonix Pharmaceuticals).
