# Supplementary material for: The Cytokine Ciliary Neurotrophic Factor (CNTF) Activates Hypothalamic Urocortin-Expressing Neurons Both In Vitro and In Vivo
Source: PLoS One. 2013 Apr 23;8(4):e61616. doi: 10.1371/journal.pone.0061616 (PMC3633986; doi:10.1371/journal.pone.0061616)
Supplement: Table S1 — Primer sequences used in the manuscript. (PDF) [file pone.0061616.s003.pdf]

Supplementary Table 1 - primer sequences used in Purser et al.

RT-PCR Primers:

Primer Name	Forward	Reverse	Annealing Temperature	Product size:
CNTF Receptor	atactgcgaagcttagaactgggc	gcacagtcacattgaaggtattgg	60 °C	214 bp
BDNF	aagctttgcggatattgcgaaggg	tgtacaagtccgcgtccttatggt	60 °C	479 bp
NT	ataggaatgaaccttcagctg	gtaggaggccctcttgagtat	60 °C	498 bp
Urocortin I	gcgtcttcagcccgtccccggggacagagt	gccgatcacttgcccaccgaatcgaatatg	60 °C	456 bp

qPCR Primers:

Primer Name	Forward	Reverse	Annealing Temperature
AgRP	cggaggtgctagatccacaga	aggactcgtgcagccttacac	60 °C
Urocortin I	gcagagcagaaccgcatcatattc	aagggaaagggtcaaggctttcgt	60 °C
Urocortin II	agcaactctaaagccagcccttac	tgattcctggcagccttgtaacga	60 °C
Urocortin III	acaagctggaagatgtgcccttgct	ccgcggcacctgagaagttct	60 °C
Histone 3a	cgcttccagagtgcagctatt	atcttcaaaaaggccaaccagat	60 °C
BDNF	aagctttgcggatattgcgaaggg	tggaacattgtggctttgctgtcc	60 °C
NT	TaqMan® Gene Expression Assay, SM: Catalogue # 4331182	Assay ID: Mm00481140_m1<br>Gene Symbol: Nts, mCG19551	60 °C
	TaqMan® Universal PCR Master Mix: Catalog # 4304437		
